# Chinese striped-neck turtles vocalize underwater and show differences in peak frequency among different age and sex groups

**DOI:** 10.7717/peerj.14628

**Published:** 2023-01-13

**Authors:** Lu Zhou, Jinhong Lei, Xiaofei Zhai, Haitao Shi, Jichao Wang

**Affiliations:** Ministry of Education Key Laboratory for Ecology of Tropical Islands, Key Laboratory of Tropical Animal and Plant Ecology of Hainan Province, College of Life Sciences, Hainan Normal University, Haikou, China

**Keywords:** Sound recording, Underwater vocalization, Call types, Peak frequency, Chinese striped-neck turtles, *Mauremys sinensis*, Sex difference

## Abstract

**Background:**

Turtle vocalizations play an important role throughout their lives by expressing individual information (position, emotion, or physiological status), reflecting mating preferences, and synchronizing incubation. The Chinese striped-neck turtle (*Mauremys sinensis*) is one of the most widely distributed freshwater turtles in China, whose wild population is critically endangered. However, its vocalization has not been studied, which can be the basis for behavioral and ecological studies.

**Methods:**

Five different sex–age groups of turtles were recorded underwater in a soundproof room. Cluster analysis and principal component analysis for classification of Chinese striped-neck turtle calls were unreasonable. The turtle calls were manually sought using visual and aural inspection of the recordings in Raven Pro 1.5 software and classified according to differences perceived through auditory inspection and the morphological characteristics of the spectrograms. The results of similarity analysis verified the reliability of manual classification. We compared the peak frequency of the calls among different age and sex groups.

**Results:**

We identified ten *M. sinensis* call types, displayed their spectra and waveforms, and described their auditory characteristics. Most calls produced by the turtles were low-frequency. Some high-frequency call types, that are common in other turtle species were also produced. Similar to other turtles, the Chinese striped-neck turtle generates harmonic vocalizations. Courtship behaviors were observed when one of the call types occurred in the mixed-sex group. Adult females produced more high-frequency call types, and subadult males had higher vocalizations than other groups. These results provide a basis for future research on the function of vocalizations, field monitoring, and conservation of this species.

## Introduction

Sound is one of the most common forms of communication among animals (Gerhardt & Huber, 2002) and is important in information transmission for species living in dark environments or underwater (Shang, 2014). Turtle vocalizations play an important role throughout their lives by expressing individual information (size and health status) (Sacchi et al., 2003), reflecting the sexual preference of female turtles (Galeotti et al., 2005a), and influencing mating success (Sacchi et al., 2003). Moreover, vocalization among hatchlings in a nest is related to the synchronization of emergence (Ferrara, Mortimer & Vogt, 2014; Monteiro et al., 2019).

Freshwater turtle vocalizations have been evaluated for many species and those studies provided a foundation for acoustic studies of turtles; however, the vocalizations of Mauremys have not been recorded (Ferrara et al., 2014a; Chen & Wiens, 2020; Jorgewich-Cohen et al., 2022; Zhou et al., 2022). Vocalizations were recorded in the wild or semi-wild and manually classified based on visual and auditory differences (Giles et al., 2009; Ferrara et al., 2014b; Ferrara et al., 2017). Although almost all research on sea turtle vocalizations has focused on hatchling vocalizations in air (Monteiro et al., 2019; Ferrara et al., 2019), a recent study showed that free-ranging juvenile green turtles produced ten types of sounds underwater (*Chelonia mydas*) (Charrier et al., 2022).

The wild Chinese striped-neck turtle (*Mauremys sinensis*), which lives in plains and hilly areas (Zhou, 2006; Li et al., 2013), is among the most widely distributed freshwater turtles in China. Although the species can be bred in large numbers in captivity, its wild population has been declared critically endangered by the International Union for Conservation of Nature (Li, Rao & Wang, 2021). This species has been extensively studied on its taxonomy (Barth et al., 2004; Hu et al., 2013), physiology (Pan, Zhang & Ji, 2003; Zhang et al., 2011; Blasio et al., 2021), molecular biology (Feldman & Parham, 2004; Fong et al., 2007; Fong & Chen, 2010) and ecology (Bour, 2007; Chen & Lue, 2010; Duong, Ngo & Nguyen, 2014; Wang et al., 2020a; Wang et al., 2020b), but its bioacoustics have not been evaluated. *Chinese striped-neck turtle* prefers to reside in deep and slow-current pools and has a highly aquatic nature (Chen & Lue, 2008); thus, underwater vocalization may be crucial for communication among individuals. Therefore, vocalization research can provide a basis for further behavioral and ecological studies of *M. sinensis*.

This study was conducted to verify whether Chinese striped-neck turtles emit sounds underwater using passive acoustic monitoring and to describe the features of these sounds. We also classified the sounds and analyzed differences in vocalization among different sex and age groups.

## Materials & Methods

### Data collection

**Figure 1:**
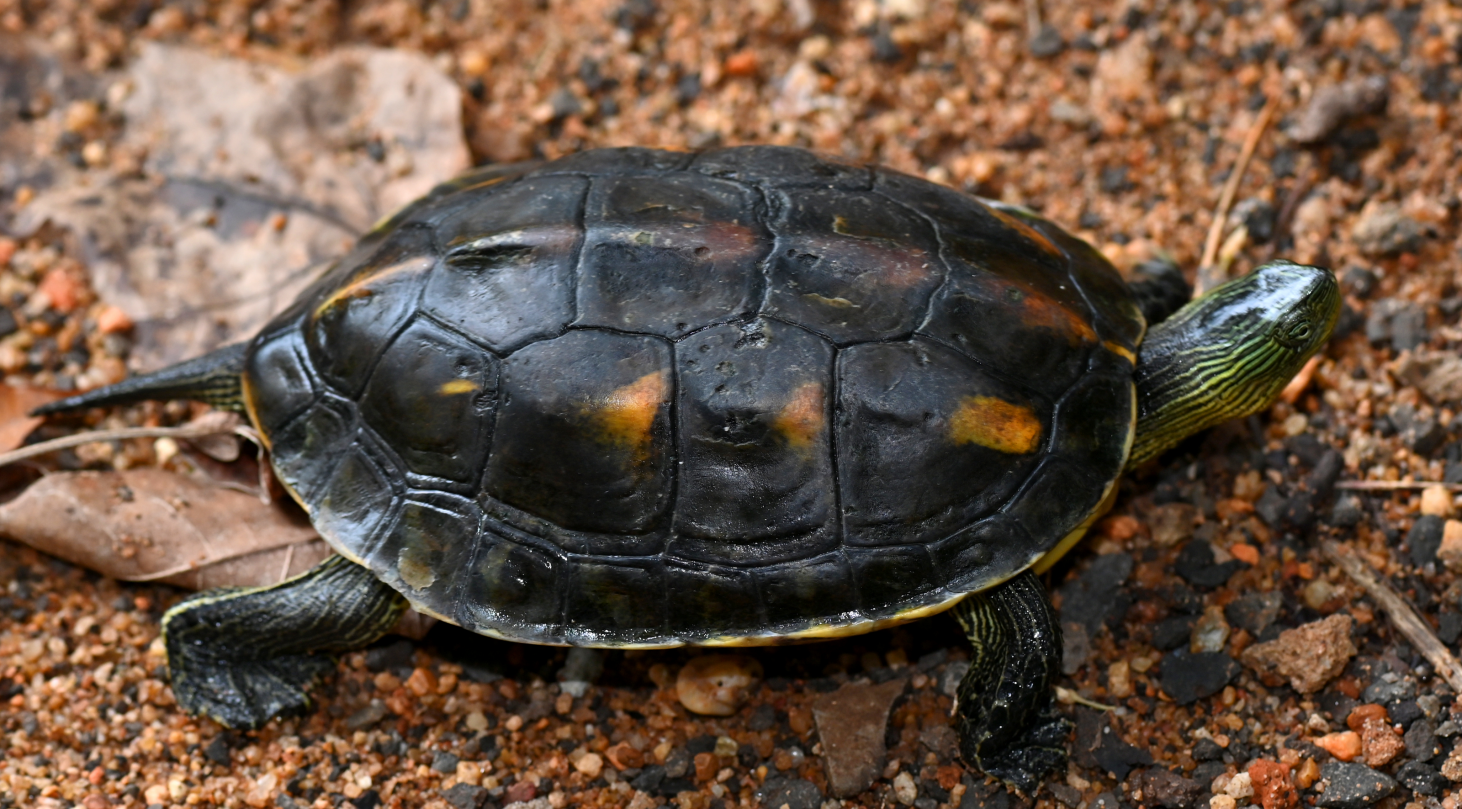
A photo of a Chinese striped-neck turtle. Photo credit: Lu Zhou.

To distinguish the call differences among turtles of different sexes and ages, we obtained 16 healthy captive Chinese striped-neck turtles (Fig. 1) from a turtle farm and divided them into four groups according to their age and sex to conduct recordings. The groups included, respectively: four adult females, four adult males, four subadult females, and four subadult males. We mixed all adult turtles (*n* = 8) and recorded their vocalizations. Five groups of recordings were obtained. To diminish environmental noise during the recording, the turtle vocalizations were recorded in a soundproof room at the College of Life Sciences, Hainan Normal University.

Recordings were conducted in an inflatable plastic circular cistern (diameter of 1.2 m and water depth of 30 cm), in which the turtles were free to move. The hydrophone was suspended 15 cm below the water surface at the center of the tank. An underwater sound recorder, the Song Meter SM4 (Wildlife Acoustics, Inc., Maynard, MA, USA), was used to collect turtle calls (sampling rate: 44,100 Hz; sensitivity: −165 dB Re: 1 V/µPa; omnidirectional hydrophone bandwidth: 2 –30,000 Hz ± 3 dB; gain: 16 dB). The hydrophone had been calibrated by the company (Wildlife Acoustics, Inc.) before purchase. Recording was performed in mid-June 2021. Each group was continuously recorded for 10 h, from 9:00 to 19:00, giving a total of 50 h of data. During the recording, the turtles were monitored *via* closed-circuit television to avoid human interference and ensure that they remained in the water. No turtles were excluded from the experiment. The average sound pressure level (SPL) of the underwater background noise (without turtles in water) in the tank was 92.35 dB. The energy of background noise was mainly concentrated at low frequencies below 50 Hz. Noise below 50 Hz had a SPL of 70 dB, and as the frequency reaches above 200 Hz, the SPL of noise drops to approximately 40 dB. All authors were aware of the group allocation at the different stages of the experiment. Because we performed passive acoustic monitoring experiments and not intervention experiments, and factor analysis was not required, no control group was included in this study.

After the recording, the 16 turtles were released into a semi-natural pool in the ecological park of Hainan Normal University. Animals in ecological park are regularly fed and maintained by professional staff.

### Data analysis

Acoustic data was extracted using Raven Pro 1.5 software (The Cornell Lab of Ornithology, Cornell, NY, USA). We used two methods to classify turtle calls. The first is machine classification using cluster analysis and principal component analysis (PCA), which are based on the acoustic parameters of the sound. All analysis was performed in R software version 4.1.1 (R Core Team, 2021). “Flexclust” package was used for cluster analysis (Leisch & Gruen, 2006), and PCA used the built-in package “stats” of R software (R Core Team, 2021). The second is manual classification. One researcher with experience in aquatic vocalizations manually sought the turtle sounds through visual and aural inspection of the recordings, and three other researchers classified these calls independently to ensure the repeatability of classification. Sounds with similar characteristics (spectra shape; auditory) as published turtle sounds (Giles et al., 2009; Ferrara et al., 2017) and that clearly sound like animal calls were selected. Only signals whose spectrograms displayed a high signal-to-noise ratio were selected for further acoustic analyses. The selected sound signals were classified according to differences perceived *via* auditory sensing and the morphological characteristics of the spectrograms (Giles et al., 2009; Zhou et al., 2022). When two signals showed the same auditory characteristics and extremely similar spectral morphologies, they were classified as the same type; otherwise, they were defined as different types. The recordings were monitored using earphones (IE300; Sennheiser, Hanover, Germany).

The sound characteristics of low frequency (Hz), high frequency (Hz), peak frequency (Hz), signal duration (ms), and the number of harmonics and inflections were extracted using the Raven Pro 1.5 software. The sound pressure level (SPL) of the calls was calculated using MATLAB R2017a (MathWorks, Natick, MA, USA) based on the following formula (Au & Hastings, 2008).

\begin{eqnarray*}& & \mathrm{SPL}=20~{\mathrm{log}}_{10}(p/{p}_{\mathrm{ref}})(\mathrm{dB}) \end{eqnarray*}

where *p* is the sound pressure and *p*_ref_ is the underwater reference sound pressure.

Because turtles always make sounds when they crawl and move underwater, we identified these sounds prior to analyzing the vocalizations. Turtle behaviors were observed during the recording of underwater sounds from the tank. We next analyzed the frequency spectra and auditory characteristics of the sounds produced by different behaviors using Raven Pro 1.5 software. Subsequently, we identified the corresponding sounds produced by common turtle behaviors in water: crawling, stroking water, releasing bubbles, sucking water, scratching claws against the tank bottom, rubbing turtle shells against the tank wall, rubbing turtle shells against the hydrophone, colliding turtle shells, and scratching the tank wall with claws. The spectrograms and waveforms of these sounds are shown in Fig. S1.

### Statistical analyses

To test the reliability of the manual classification, we examined the similarity between signals of different call types and similarity between signals of the same call type (Manolakis, Ingle & Kogon, 2005; Zheng, 2011). Pearson’s linear correlation coefficient (Gibbons, 1985) was used to detect similarities. The calculation was performed in MATLAB R2021a (MathWorks, Inc., Natick, MA, USA).

The Kolmogorov–Smirnov test (Dallal & Wilkinson, 1986) was used to estimate whether the peak frequency of the sounds in each group followed a normal distribution. The least significant difference test (post hoc test algorithms) was used to evaluate the differences in the call peak frequency between each of the two groups when the distribution was normal (*p* > 0.05). If the distribution was non-normal (*p* < 0.05), Kruskal–Wallis analysis (non-parametric tests algorithms) (Siegel, 1956) was used to examine differences among the five groups. If the difference among the five groups in Kruskal–Wallis analysis was significant (*p* < 0.05), then pairwise comparisons (difference between each of the two groups) was calculated; if the difference among the five groups was insignificant (*p* > 0.05), pairwise comparisons were not conducted. Differences were considered significant at *p* < 0.05. All analyses were performed using SPSS Statistics 20.0 (SPSS, Inc., Chicago, IL, USA).

### Ethics note

All applicable international, national, and/or institutional guidelines for the care and use of animals were followed. All procedures performed in studies involving animals were approved by the Animal Research Ethics Committee of Hainan Provincial Education Centre for Ecology and Environment, Hainan Normal University (No. HNECEE-2021-002).

## Results

**Figure 2:**
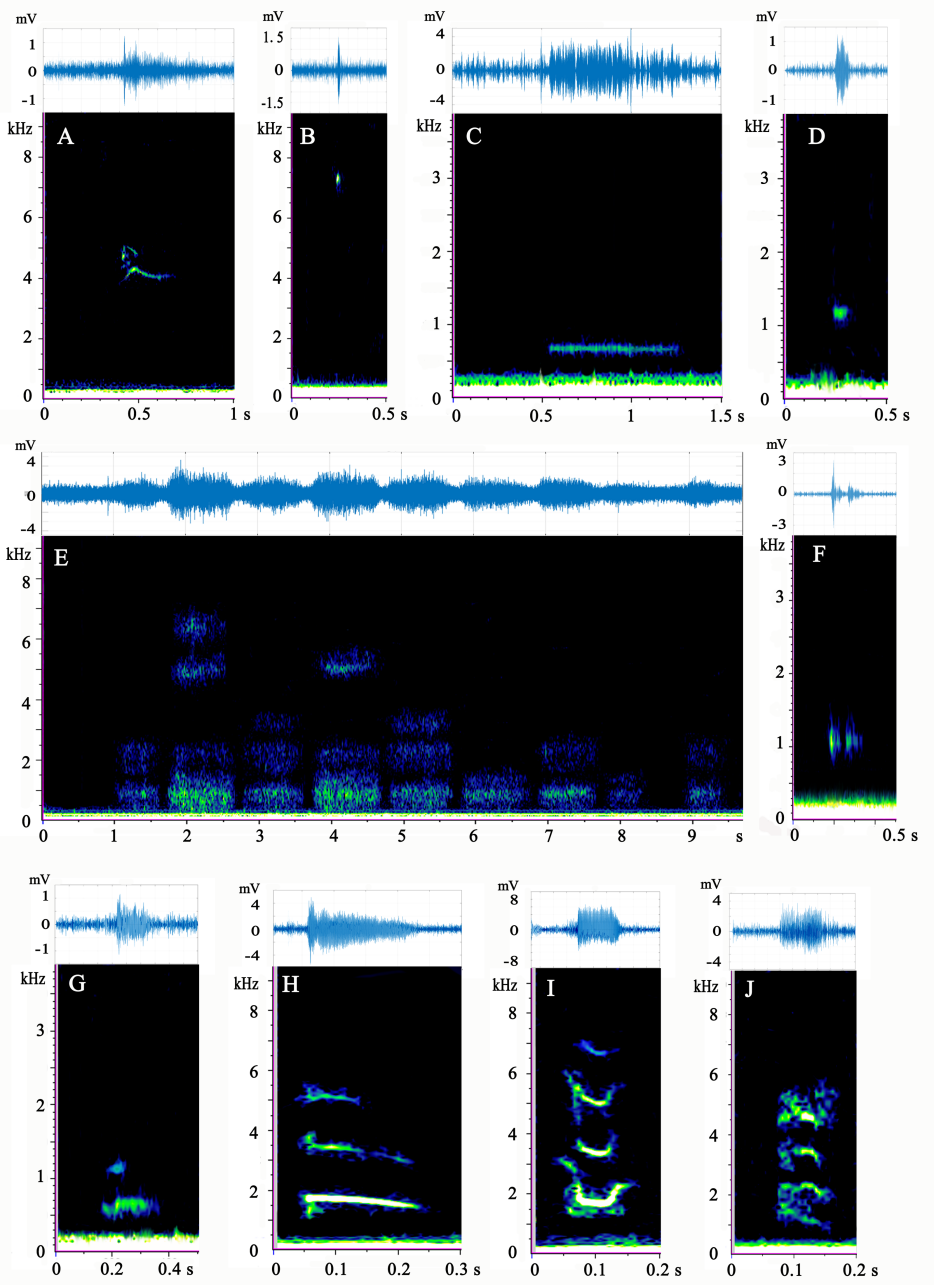
(A–J) Spectrogram and waveform views of the sounds produced by *Mauremys sinensis*. All spectrograms were obtained by Raven Pro 1.5 using Hamming windows with 512 pt FFT. The waveforms were drawn by MATLAB R2017b.

A total of 860 distinguishable calls were detected while recording the 16 *M. sinensis* individuals (Table 1). Of these, 435 calls were made by females, and 234 were made by males. Cluster analysis clearly divided all calls into eight groups, but a large number of different call types were grouped together (Fig. S2), for example, completely different calls (harmonics, high-frequency pulses, and long-duration waves) were grouped together. PCA was unable to subdivide sounds very well, because most of the calls were assembled together (Fig. S3). Therefore, the results of PCA and clustering were unreasonable. Turtle calls were assigned to ten types manually based on the aural character, frequency, and morphology of the spectrum (Fig. 2).

### Description of call types

The spectrograms and waveforms of each call type are shown in Fig. 2. The acoustic parameters of each type are shown in Table 2. Audio samples of each type are available in Audio S1–S10.

**Table 1:** The number and proportion of call types produced by *Mauremys sinensis* in each group.

**Type**	**Adult females**	**Ratio (%)**	**Adult males**	**Ratio (%)**	**Subadult females**	**Ratio (%)**	**Subadult males**	**Ratio (%)**	**Mixed-sex**	**Ratio (%)**	**Sum**
**A**	43	36.13	9	7.56	9	7.56	47	39.50	11	9.24	119
**B**	26	78.79	2	6.06	1	3.03	2	6.06	2	6.06	33
**C**	61	22.59	85	31.48	50	18.52	25	9.26	49	18.15	270
**D**	113	53.81	23	10.95	40	19.05	30	14.29	4	1.90	210
**E**	54	31.95	1	0.59					114	67.46	169
**F**	17	60.71	1	3.57	6	21.43	2	7.14	2	7.14	28
**G**	10	50.00	1	5.00	3	15.00	5	25.00	1	5.00	20
**H**					1	12.50			7	87.50	8
**I**			1	50.00	1	50.00					2
**J**									1	100.00	1
Total	324	37.67	123	14.30	111	12.91	111	12.91	191	22.21	860

Type A is a high-frequency call, consisting of 1–3 harmonics, most of which are a single harmonic with 1–2 inflection points. It has an “M” or “inverted V” spectrum shape, which sounds like the short squeak of a chick.

Type B is a single high-frequency click with a narrow band and sounds like a short chirp. This sound type occurred at a very high ratio in adult female group.

Type C is a low-frequency call with a relatively long duration. It involves a horizontal line on the spectrum and sounds like a ship’s whistle. It was the most common call type of the species and appeared evenly in each group.

Type D is a low-frequency call that resembles a circular spot on the spectrum and has a loud, short “beep” sound. More than half of the vocalizations of this type were produced by the adult female group.

Type E has a cloud-like appearance on the spectrum and occurs in different frequency bands. Typically, several clouds occupied the high, medium, and low frequencies simultaneously. This type often occurred as several calls in a row (1–15) and sounded similar to a very heavy, loud gasp. This pattern occurred most frequently in the mixed-sex group. Male courtship behavior toward females was observed before and during type E vocalization. The male climbed directly in front of the female and faced her, craned his neck to get his head close to her snout, and then moved his head from one side of her snout to the other and back again several times.

Type F is composed of two parts in the low-frequency band: one part before the band and another after it. This call type sounds like a strong, echoing creak and emitted 28 times, mostly by the adult female group.

Type G has two harmonics that sound similar to a loud dog bark. Vocalizations of this type were rare and mostly occurred in the adult female group.

Type H is the sweep of three harmonics without an inflection point on the spectrum, which sounds like a long, falling chirp. This call type was very rare and was mostly produced by the mixed-sex group.

Type I sounds like a chick crow that descends and then ascends. Its spectrogram consists of three or four “U”-shape harmonics with two inflection points. This call occurred only in the adult male and subadult female groups.

Type J consists of four curved harmonics with three inflection points and sounds similar to the call of a house rat. Only one sample of this call was recorded, which occurred in the mixed-sex group.

**Table 2:** Descriptive statistics of the acoustic parameters of each call type.

**Type**	**Low frequency (Hz) mean ± SD**	**High frequency (Hz) mean ± SD**	**Duration (ms) mean ± SD**	**Peak frequency (Hz) mean ± SD**	**Frequency range (Hz)**	**No. of harmonics**	**Number of inflection points**	**Sound pressure level (dB) mean ± SD**
A	6,412 ± 3,054	6,965 ± 3,152	117 ± 77	6,714 ± 3,092	1,269–14,007	1–3	1–2	96.07 ± 2.87
B	9,937 ± 3,980	10,489 ± 4,221	37 ± 28	10,205 ± 4,057	3,161–17,233	–	–	94.29 ± 1.65
C	558 ± 512	704 ± 543	324 ± 237	631 ± 519	99–6,192	–	–	106.16 ± 8.75
D	807 ± 410	975 ± 431	57 ± 54	891 ± 411	152–2,600	–	–	97.77 ± 7.17
E	1,776 ± 2,077	3,804 ± 2,151	468 ± 210	2291 ± 2,059	128–8,356	1–3	–	97.68 ± 4.76
F	799 ± 292	1,007 ± 337	74 ± 67	903 ± 309	231–1,605	–	–	96.04 ± 1.92
G	521 ± 310	971 ± 403	109 ± 53	613 ± 340	130–1,957	2	–	95.98 ± 2.73
H	841 ± 440	2,056 ± 1,357	197 ± 83	999 ± 508	238–5,236	3–4	–	102.03 ± 5.03
I	501 ± 280	3,361 ± 3,642	250 ± 209	560 ± 213	303–5,936	3–4	2	100.53 ± 6.80
J	758	5557	88	4,565	758–5,557	4	3	96.53

### Similarity calculation

The average correlation coefficient among the ten call types was 0.22 and that among the internal signals of each call type was 0.88. The specific correlation coefficients are shown in Table 3. According to the results, the similarity between each call type was small and the difference was large, whereas the signal similarity within each type was large and the difference was small. Thus, the results of manual classification were reliable.

**Table 3:** Average value of correlation coefficients for signals within each call type and between call types.

	Call Types	A	B	C	D	E	F	G	H	I	J
Average value of correlation coefficients between call types	A										
		B	0.19									
		C	0.28	0.23								
		D	0.40	0.37	0.12							
		E	0.24	0.23	0.36	0.24						
		F	0.28	0.25	0.11	0.23	0.21					
		G	0.23	0.33	0.13	0.15	0.21	0.11				
		H	0.32	0.29	0.14	0.18	0.14	0.11	0.14			
		I	0.26	0.29	0.29	0.22	0.11	0.25	0.17	0.15		
	J	0.28	0.29	0.28	0.24	0.19	0.19	0.15	0.14	0.23	
Average value of correlation coefficients within call types		0.72	0.87	0.90	0.95	0.70	0.84	0.80	0.73	0.89	–

**Notes.**

“–” represents that the similarity calculation cannot be performed because the sample size was less than 2.

### Differences in peak frequency of overall vocalization between sex-age groups

Based on the results of the distribution test, the data from the five groups showed a non-normal distribution (adult females: *p* < 0.001, *Z* = 0.314, *df* = 324; adult males: *p* < 0.001, *Z* = 0.362, *df* = 123; subadult females: *p* < 0.001, *Z* = 0.308, *df* = 111; subadult males: *p* < 0.001, *Z* = 0.279, *df* = 111; mixed sex: *p* < 0.001, *Z* = 0.328, *df* = 191). Therefore, a non-parametric test (Kruskal–Wallis test) was used for pairwise comparison. The peak frequency of calls emitted by adult females and subadult males was higher than that of the adult males, subadult females, and the mixed-sex groups (*p* < 0.05). The peak frequency of the mixed-sex group was higher than that of the subadult female group (*p* < 0.05) (Table 4). There was no significant difference in the peak frequency between adult males and subadult females, between adult males and the mixed-sex group, and between adult females and subadult males (*p* > 0.05).

**Table 4:** Difference analysis of the calls in peak frequency between groups.

*P H*	Adult females	Adult males	Subadult females	Subadult males	Mixed-sex adults
Female adults	−	0.000	0.000	0.182	0.000
Male adults	6.037	−	0.333	0.000	0.433
Female subadults	8.346	2.128	−	0.000	0.000
Male subadults	−2.361	−6.867	−8.773	−	0.000
Mixed sex	4.448	−2.021	−4.291	5.575	−
Peak frequency (Hz) mean ± SD	2,768 ± 3,375	1,156 ± 1,580	840 ± 782	4,752 ± 4,551	1,424 ± 1,713
*N*	324	123	111	111	191

**Notes.**

The bottom left part of the table is *H* (the standard test statistic), the upper right part of the table is *P* (the 2-slided *p*-value).

### Differences in call types among the five groups of different sexes and ages

**Figure 3:**
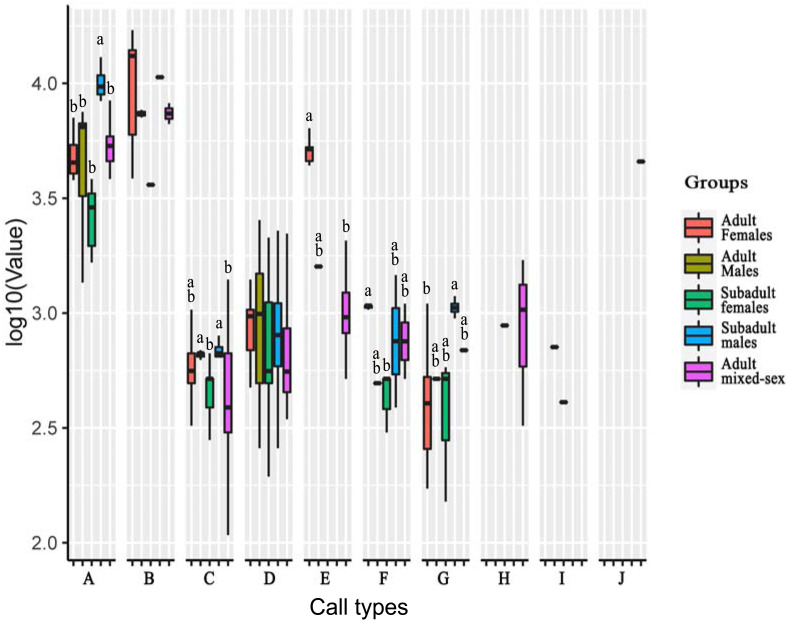
Analysis of differences in peak frequencies among five age–sex groups for all call types. The *y*-axis is the log-transformed value of peak frequency. The *x*-axis represents the call types. Boxplots with different lowercase letters refer to Kruskal–Wallis tests that yielded significant results (*p* < 0.05), and the relationship among the letters is a > b. Boxplots that share the same lowercase letters refer to no significant difference (*p* > 0.05). Call types without letters on the boxplots show no significant differences among age–sex groups.

Of the ten call types, five showed significant different frequencies among the age–sex groups. The other call types showed no significant differences in their peak frequencies among groups (Table 5; Fig. 3).

**Table 5:** Difference analysis of peak frequencies for 10 call types between age–sex groups.

**Kruskal-Wallis tests (*p*-value)**		**Call types**									
		**A**	**B**	**C**	**D**	**E**	**F**	**G**	**H**	**I**	**J**
Adult females	Adult males	1.000	*H* = 4.977, *N* = 33, *df* = 4, *P* = 0.290	0.165	*H* = 8.483, *N* = 210, *df* = 4, *p* = 0.075	1.000	0.988	1.000	−	−	−
		Subadult females		0.096			0.230		−	0.025	1.000	−	−	−
		Adult females		0.000			0.102			1.000	0.032	−	−	−
	Mixed sex	1.000				0.723		0.000	1.000	1.000	−	−	−
Adult males	Adult females	1.000				0.165		1.000	0.988	1.000	−	−	−
		Subadult females		0.096			0.000		−	1.000	1.000	−	−	−
		Adult females		0.006			1.000		−	1.000	1.000	−	−	−
	Mixed sex	1.000				0.000		0.899	1.000	1.000	−	−	−
Subadult females	Adult females	0.096				0.230		−	0.025	1.000	−	−	−
		Adult males		0.248			0.000		−	1.000	1.000	−	−	−
		Adult females		0.000			0.000		−	1.000	0.232	−	−	−
	Mixed sex	0.519				1.000		−	1.000	1.000	−	−	−
Adult females	Adult females	0.000				0.102		−	1.000	0.032	−	−	−
		Adult males		0.006			1.000		−	1.000	1.000	−	−	−
		Subadult females		0.000			0.000		−	1.000	0.232	−	−	−
	Mixed sex	0.000				0.001		−	1.000	1.000	−	−	−
Mixed sex	Adult females	1.000				0.723		0.000	1.000	1.000	−	−	−
		Adult males		1.000			0.000		0.899	1.000	1.000	−	−	−
		Subadult females		0.519			1.000		−	1.000	1.000	−	−	−
	Adult females	0.000		0.001		−	1.000	1.000	−	−	−

**Notes.**

“ *p* < 0.05” indicates that there is a significant difference between groups; otherwise, there is no significant difference between groups; “ −” indicates that comparisons cannot be made because there are less than two individual calls of that type in a group. Types B and D were not conducted paired comparison because there is no significant difference in the five groups for these two call types ( *p* > 0.05).

For Type A, the peak frequency of subadult males was higher than that of all other groups (*p* < 0.05). There were no significant differences among the other groups (*p* > 0.05).

For Type C, the peak frequency of adult males and subadult males was significantly higher than that of subadult females and the mixed-sex group (*p* < 0.05). There were no significant differences among adult females and all other groups, between subadult females and the mixed-group, and between adult males and subadult male (*p* > 0.05).

For Type E, the peak frequency of adult females was significantly higher than that of the mixed-group (*p* < 0.05). Differences could not be calculated for the other groups because of the small number of vocal samples for this call type.

For Type F, the peak frequency of adult females was higher than that of subadult females (*p* < 0.05). There were no significant differences among the other groups (*p* > 0.05).

For Type G, the peak frequency of subadult males was higher than that of adult females (*p* < 0.05). There were no significant differences among the other groups (*p* > 0.05).

## Discussion

### Vocalization characteristics

Natural selection of both the producer and receiver helps to reduce the misinterpretation of conspecific signals (Heyer, Garcia-Lopez & Cardoso, 1996). Utilization of strict intraspecies signaling to prevent mismatching has been documented in sympatric species of anurans and birds (Pfennig, 1998; Wollerman & Wiley, 2002; Höbel & Gerhardt, 2003). Chinese striped-neck turtles produce a variety of vocalizations, and strict signal exchange might be beneficial for reducing interspecies mismatching and for helping individuals to identify members of the same species, as other freshwater turtles reside in its habitat (Zhou, 2006).

Chinese striped-neck turtles emit two types of high-frequency calls (Type A and Type B). Although previous studies showed that the hearing sensitivity of turtles is concentrated at low frequencies (Christensen-Dalsgaard et al., 2012; Piniak et al., 2012a; Piniak et al., 2012b; Wang et al., 2019), several aquatic turtle species have been found to produce high-frequency calls (that can reach 6,000–13,000 Hz) (Ferrara, Vogt & Sousa-Lima, 2013; Ferrara et al., 2019; McKenna et al., 2019; Monteiro et al., 2019). Natural rivers or lakes contain large amounts of low-frequency noise, whereas the high-frequency energy is relatively low (Wang et al., 2020a; Wang et al., 2020b; Zhou et al., 2021). High-frequency calls help to resist the interference from low-frequency noise in communication. There is a lack of hearing research on Chinese striped-neck turtles; thus, further studies are needed to determine whether the turtles can hear their own high-frequency signals, or if it is just a potential functional signal redundancy as observed for some of the vocalizations of *Alligator sinensis* (Todd, 2007; Wang et al., 2007; Wang et al., 2009). Snakes can produce sounds up to 10 kHz, which is well above their hearing range; however, the low level of acoustic specialization within the sounds produced by snakes and the low potential for encoded information content indicate limited communication capabilities (Young et al., 1999; Young, 2003). It was reported that *Ptyas mucosus* may be an acoustic Batesian mimic of the king cobra, *Ophiophagus hannah* (Young, Solomon & Abishahin, 1999). Acoustic communication evolved independently in most major tetrapod groups and is strongly conserved over time, whereas the role of ecology in shaping signal evolution applies to deep timescales (Chen & Wiens, 2020). Therefore, the various high-frequency call types of Chinese striped-neck turtles may be the result of a combination of evolution and ecology.

Chinese striped-neck turtles produced three call types with harmonics (Types H, I and J). Vocalizations with similar spectra can be found in many species, such as tortoises (Campbell & Evans, 1972; Sacchi et al., 2003; Galeotti et al., 2005b), sea turtles (Ferrara et al., 2014c; Ferrara, Mortimer & Vogt, 2014; Ferrara et al., 2019; Monteiro et al., 2019), and freshwater turtles (Giles et al., 2009; Ferrara, Vogt & Sousa-Lima, 2013; Ferrara et al., 2014b; Ferrara, Vogt & Pappas, 2018). The ability to produce these three call types may be related to the structure of turtles’ laryngeal vocal organ, which comprises three cartilages and two pairs of muscles, along with two diverticula supported by the cricoid that may function as a low-frequency resonating chamber, improving the harmonic structure of turtle calls (*e.g.*, Testudinidae) (Sacchi et al., 2004). Although the larynx of turtles is the most differentiated and variable among reptiles (Siebenrock, 1899), the larynx of turtles (including sea turtles, freshwater turtles and tortoises) is cradled by the broad plate constituting the body of the hyoid (Siebenrock, 1898) and forming a dorsomedial cricoid cartilage (Göppert, 1937; Russell & Bauer, 2021). These common structures may be the reason why many turtles produce similar calls. As the calls were recorded in an artificial environment, not all acoustic descriptions reflect field recordings, particularly the call duration (Giles et al., 2009).

### Vocalizations probably associated with courtship

We think that Type E is associated with courtship in Chinese striped-neck turtles. The recordings were performed during the breeding season of the turtles (from February to June) (Zhou, 2006). Type E mostly was produced by the mixed-sex group (67.46%). We observed male courtship behavior toward females before and during Type E vocalization. Typically, the male climbed directly in front of the female and faced her, then craned his neck so that his head was close to her snout, and then moved his head from one side of her snout to the other and back again several times. We found that female turtles responded in three ways: repeating the movement, not moving, or turning away. No back-climbing mating behavior was observed throughout the monitoring process. We could not determine whether Type E was emitted by a female or a male in the video. On the other hand, Type E often occurred as several successive calls and resembled a heavy gasp. Several turtle species produced trains of harmonics when courting, mating and nesting, such as leatherback (*Dermochelys coriacea*) (Ferrara et al., 2014c), *Geochelone carbonaria* (Campbell, 1967), marginated tortoises (*Testudo. marginata*) (Sacchi et al., 2003), and Hermann’s tortoises (*T. hermanni*) (Galeotti et al., 2005a).

### Sex differences in vocalization

The peak frequency of subadult males was higher than those of the other groups. Disparate vocalization in a species is often caused by sex differences in the structure of the vocal organs (Tobias & Kelley, 1987; Sathyan, Engelbrecht & Couldridge, 2017). A larger body size usually indicates a larger vocal organ, which produces lower frequencies (Narins, Feng & Fay, 2006; Garcia et al., 2013). Studies on tortoises showed that vocal frequency is inversely proportional to the animal size (Galeotti et al., 2005b). This may explain why subadult males (had the smallest body size among groups) emitted higher-frequency vocalizations compared to those of the other groups.

In our recordings, the call peak frequency did not differ between the adult male and adult female groups. However, the high-frequency call types were mostly produced by adult females, which cannot be explained by inverse proportionality to the animal size because adult females had the largest body size among all groups. First, body size does not always reflect the vocal organ size. In many animals, large females produce higher calls compared to smaller males, such as owls (Miller, 1934), jacanas (Buck et al., 2020), chimpanzee (*Pan troglodytes*) (Ammie, 2019), baboons (*Papio cynocephalus*) (Fischer et al., 2004) and gibbons (*Hylobates lar*) (Barelli et al., 2013). In addition, sex differences in vocalizations may arise for various reasons, such as the way a sound is produced (Lockner & Youngren, 1976), different socio-sexual strategies (Ammie, 2019), and sexual dimorphism in auditory perception (Shen et al., 2020; Zhang et al., 2021). Differences in voice can reflect sex specificity and individual markers (Grawunder et al., 2018). Chinese striped-neck turtles live in groups (Chen & Lue, 2008). Females produced more high-frequency calls compared to males, possibly because of the differences in their home area sizes (8.59 hm^2^ for females and 2.46 hm^2^ for males) (Ma et al., 2013). High-frequency sound is more penetrating and not easily masked by low-frequency environmental noise in a large home area. The reasons for the vocalization differences of Chinese striped-neck turtles should be confirmed in further behavioral experiments and physiological studies.

### Method of classification

Classifying sounds can be difficult and time- consuming because of acoustic variation across environments and individuals (Keen et al., 2021). When the amount of data is not very large, many scholars choose manual division (Giles et al., 2009; Ferrara et al., 2014b; Ferrara et al., 2017). Machine learning offers an objective approach for detecting and distinguishing vocal signals (Acevedo et al., 2009; Stowell et al., 2019). However, this approach requires first manually classifying elements or vocalizations, a process that can be subjective. Unsupervised machine learning approaches do not require a labeled training dataset (Valletta et al., 2017), but they require quantifying numerous acoustic parameters, which also adds a lot of work (Keen et al., 2021). In our study, neither cluster analysis nor PCA classification could accurately distinguish different types of calls, probably because we only extracted several acoustic parameters which are not enough for the classification. Piczak (2015) verified that trained and attentive listeners can achieve greatly high levels of accuracy when classifying a large dataset. Calculating the similarities among sounds is a common way to check whether different sounds belong to a same type (Manolakis, Ingle & Kogon, 2005; Zheng, 2011; Zhou et al., 2022). Although we finally chose the manual classification, we added the similarity calculation to prove its reliability.

## Conclusions

We identified ten call types of *M. sinensis*, displayed their spectra and waveforms, and described their auditory characteristics. Courtship behavior was observed when Call Type E occurred in the mixed-sex group. Moreover, adult females produced more high-frequency call types, and subadult males had higher vocalizations than other groups. However, we did not verify the functions of the turtle vocalizations, and the reasons for vocalization differences should be confirmed in further behavioral experiments and physiological studies. These results provide a basis for additional research of the function of vocalizations, field monitoring, and conservation of this species.

##  Supplemental Information

10.7717/peerj.14628/supp-1 Data S1 Raw data of every call’s acoustic parameters of each group and their catagory

10.7717/peerj.14628/supp-2 Dataset S1 Audio of Mauremys sinensis vocalizations The dataset included the underwater vocalizations of five groups of Mauremys sinensis of different ages and sexes. The folder for each group contains different call types sub-folders.

10.7717/peerj.14628/supp-3 Figure S1 The spectrograms and waveforms of cramling and moving noise of *Mauremys sinensis* a, crawling; b, stroking water; c, releasing bubbles; d, sucking water; e, scratching claws against the tank bottom; f, rubbing turtle shells against the tank wall; g, rubbing turtle shells against the hydrophone; h, colliding turtle shells; i, sscratching the tank wall with claws. The pictures were obtained by Raven Pro 1.5 software.

10.7717/peerj.14628/supp-4 Figure S2 Cluster analysis on the calls of *Mauremys sinensis*

10.7717/peerj.14628/supp-5 Figure S3 PCA analysis on the calls of *Mauremys sinensis*

10.7717/peerj.14628/supp-6 Supplemental Information 1 ARRIVE 2.0 Checklist

10.7717/peerj.14628/supp-7 Audio S1 Audio of Call type A This is an audio sample of Call type A, and the inverted V shape between 3.5 KHz and 5 KHz is the signal.

10.7717/peerj.14628/supp-8 Audio S2 Audio of Call type B This is an audio sample of call type B, and the light spot between 7 KHz and 8 KHz is his signal.

10.7717/peerj.14628/supp-9 Audio S3 Audio of Call type C This is an audio sample of Call type C, and the liminous line between 500 Hz and 1 KHz is the signal.

10.7717/peerj.14628/supp-10 Audio S4 Audio of Call type D This is an audio sample of Call type D, and the liminous spot between 1 KHz and 1.5 KHz is the signal.

10.7717/peerj.14628/supp-11 Audio S5 Audio of Call type E This is an audio sample of Call type E, and the several columns of misty clouds are the signals.

10.7717/peerj.14628/supp-12 Audio S6 Audio of Call type F This is an audio sample of Call type F, and the group of two liminous successive spots between 0.5 KHz and 1.5 KHz is the signal.

10.7717/peerj.14628/supp-13 Audio S7 Audio of Call type G This is an audio sample of Call type G, and the column of two liminous short lines between 0.5 KHz and 1.5 KHz is the signal.

10.7717/peerj.14628/supp-14 Audio S8 Audio of Call type H This is an audio sample of Call type H, and the column of three liminous lines between 1 KHz and 6 KHz is the signal.

10.7717/peerj.14628/supp-15 Audio S9 Audio of Call type I This is an audio sample of Call type I, and the column of four liminous curves between 1 KHz and 7 KHz is the signal.

10.7717/peerj.14628/supp-16 Audio S10 Audio of Call type J This is an audio sample of Call type J, and the column of three liminous curves between 1 KHz and 6 KHz is the signal.

## Additional Information and Declarations

### Data Availability

The following information was supplied regarding data availability: The raw measurements are available in the Supplemental Files. The raw data shows all the acoustic variables of call types for different turtle groups. The last colony was used for differences analysis among groups.
