# Supplementary figures and images for: Chinese striped-neck turtles vocalize underwater and show differences in peak frequency among different age and sex groups
Source: PeerJ. 2023 Jan 13;11:e14628. doi: 10.7717/peerj.14628 (PMC9841902; doi:10.7717/peerj.14628)

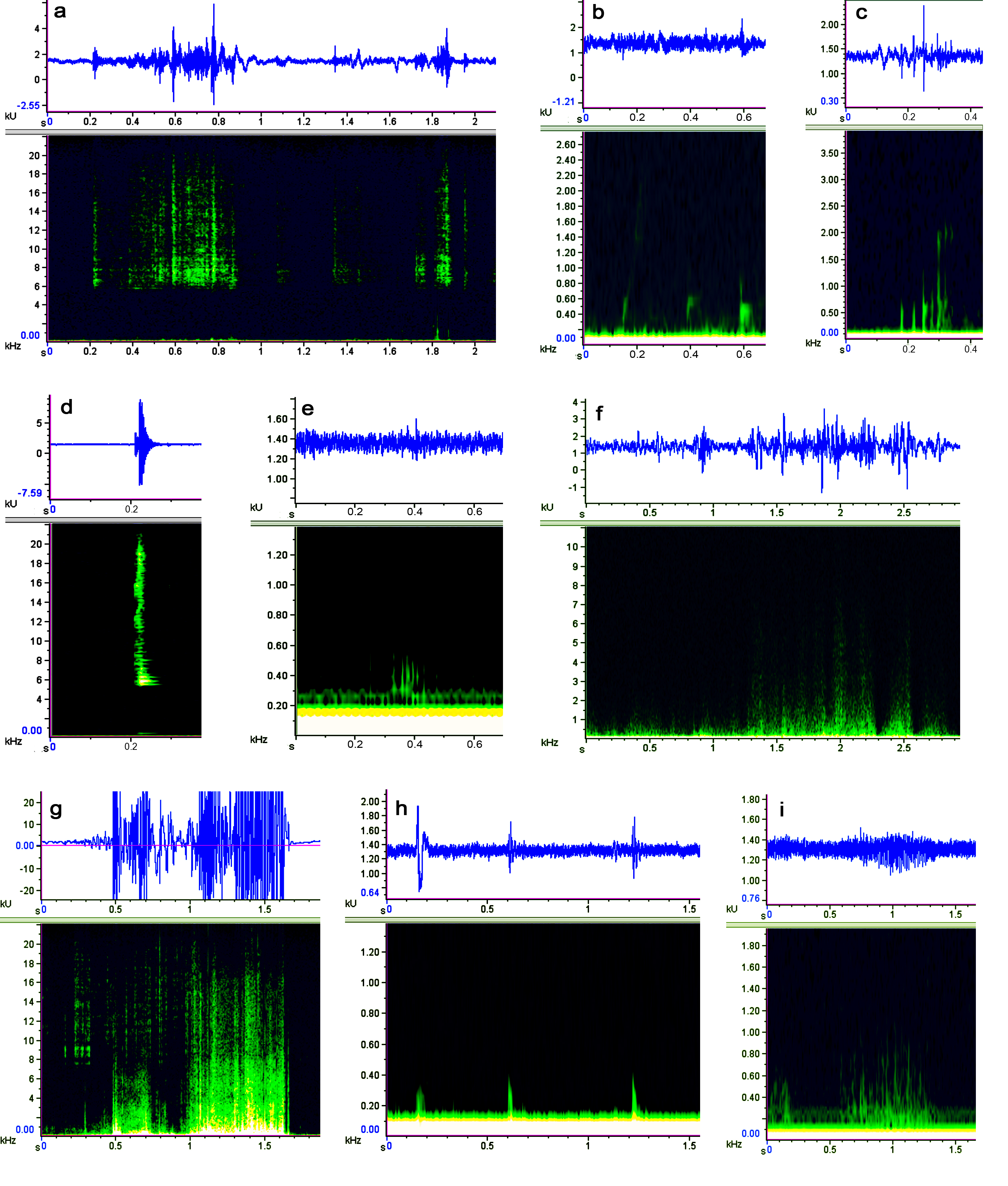

Supplement: Figure S1 — a, crawling; b, stroking water; c, releasing bubbles; d, sucking water; e, scratching claws against the tank bottom; f, rubbing turtle shells against the tank wall; g, rubbing turtle shells against the hydrophone; h, colliding turtle shells; i, sscratching the tank wall with claws. The pictures were obtained by Raven Pro 1.5 software. [file peerj-11-14628-s003.jpg]
